# Effects of Pre-, Post- and Intra-Exercise Hyperbaric Oxygen Therapy on Performance and Recovery: A Systematic Review and Meta-Analysis

**DOI:** 10.3389/fphys.2021.791872

**Published:** 2021-11-23

**Authors:** Xizhang Huang, Ran Wang, Zheng Zhang, Gang Wang, Binghong Gao

**Affiliations:** ^1^School of Physical Education and Sport Training, Shanghai University of Sport, Shanghai, China; ^2^Key Laboratory of Winter Sports Training Monitoring and Control, Heilongjiang Research Institute of Sports Science, Harbin, China

**Keywords:** hyperbaric oxygen therapy, exercise performance, meta-analysis, recovery, systematic review

## Abstract

**Background:** As a World Anti-doping Agency (WADA)-approved treatment, hyperbaric oxygen (HBO_2_) therapy has been used to improve exercise performance in sports practice.

**Objective:** We aimed to investigate the effect of pre-, post-, and intra-exercise HBO_2_ therapy on performance and recovery.

**Methods:** A literature search was conducted using EMBASE, CENTRAL, PubMed, Web of Science, and SPORTDiscus to obtain literature published until May 2021. A total of 1,712 studies that met the following criteria were identified: (1) enrolled healthy adults who were considered physically active; (2) evaluated HBO_2_ therapy; (3) included a control group exposed to normobaric normoxic (NN) conditions; (4) involved physical testing (isokinetic or dynamic strength exercise, maximal incremental treadmill/cycle exercise, etc.); and (5) included at least one exercise performance/recovery index as an outcome measure. The Cochrane risk of bias assessment tool was used to evaluate the included studies, and the heterogeneity of therapy effects was assessed using the I^2^ statistic by Review Manager 5.3.

**Results:** Ten studies (166 participants) were included in the qualitative analysis, and six studies (69 participants) were included in the quantitative synthesis (meta-analysis). In comparisons between participants who underwent HBO_2_ therapy and NN conditions, the effects of pre-exercise HBO_2_ therapy on exercise performance were not statistically significant (*P* > 0.05), and the effects of post-exercise HBO_2_ therapy on recovery were not statistically significant either (*P* > 0.05). Although individual studies showed positive effects of intra-exercise HBO_2_ therapy on exercise performance, a meta-analysis could not be performed.

**Conclusion:** Hyperbaric oxygen therapy before or after exercise had no significant effect on performance and recovery. However, hyperbaric oxygen therapy during exercise could improve muscle endurance performance, which needs to be confirmed by further empirical studies. At present, the practical relevance of these findings should be treated with caution.

## Introduction

Oxygen is an important substance for maintenance of human life activities and plays a key role in daily life, especially during exercise (Hill and Flack, 1910). To the best of our knowledge, athletes constantly need to improve their performance to continue excelling in their chosen disciplines. Thus, athletes are constantly required to perform continuous high-intensity technical movements involving shifting and changing directions during training and in competitions (Kellmann et al., 2018). The prolonged and intense oxygen utilization by tissues and organs causes hypoxia in the internal environment of the body, which greatly mobilizes the glycolysis energy supply (Carreau et al., 2011), leading to the accumulation of lactic acid in muscle or blood and depletion of glycogen in muscle (Bassett and Howley, 2000). Likewise, since oxygen delivery and utilization are influenced by hypoxia, exercise performance and recovery can lead to negative developments such as overtraining syndrome, injuries, or illnesses (Kellmann et al., 2018). Therefore, different interventions have been developed to improve performance and recovery, including nutrition therapy (Lynch et al., 2018), oxygen therapy (Sperlich et al., 2017), and cryotherapy (Rose et al., 2017). Among these, oxygen therapy has been applied as an ergogenic aid to enhance performance and accelerate recovery after exercise due to the importance of oxygen in the aerobic energy system (Hodges et al., 2003), and the low tolerance of human tissues and organs to hypoxia (Millet et al., 2010). Notably, according to the international standard prohibited list of the World Anti-doping Agency (WADA), methods for oxygen inhalation and replenishment are not classified under doping, and therefore can be applied to sports practice (Sperlich et al., 2017). With advancements in technology, the equipment and configurations used in different oxygen-therapy methods have been continuously evolving, allowing their use as auxiliary techniques in different stages of training and competition to improve the effects of training and the physical and physiological conditions of athletes as well as prevent exercise-induced fatigue.

In the field of sports science, various oxygen-therapy methods have been studied and applied to date, including hyperoxic gas supplementation, hyperbaric oxygen (HBO_2_) therapy, and micro-pressurized oxygen therapy (Sperlich et al., 2017; Ishihara, 2019). Among these, HBO_2_ therapy is defined as a treatment in which 100% oxygen is administered under a pressure greater than 1 atm absolute (ATA), and the patient breathes intermittently (Hodges et al., 2003; Branco et al., 2016). HBO_2_ therapy is not only beneficial in recovery from sports injuries and other injuries, but also contributes to an improvement in the body’s sports state and functional level (Sperlich et al., 2017; Ishihara, 2019). During long and intense training sessions, the increasingly pronounced hypoxia and the resultant variations in arterial oxygen saturation (SaO_2_) necessitate additional cardiorespiratory effort to compensate for the reduced delivery and utilization of oxygen and thereby maintain performance and muscle activity (Wolfel et al., 1991). Under these conditions, HBO_2_ therapy can increase the amount of dissolved oxygen in arterial plasma, potentially accelerating recovery (Powers et al., 1993; Peltonen et al., 1995). In addition, under conditions of exercise-induced fatigue, HBO_2_ therapy can increase the oxygen supply to the skeletal muscle system, which may activate cell activities, increase the synthesis of adenosine triphosphate, and promote the metabolic clearance of fatigue-inducing substances (Sperlich et al., 2017). Nevertheless, there is no consensus on the practical application effect of HBO_2_ therapy on performance and recovery, and the underlying mechanisms of action of this therapy require further research.

Therefore, this study systematically evaluated the effects of pre-, post-, and intra-exercise HBO_2_ therapy on performance and recovery, critically summarized the studies on the use of HBO_2_ therapy to improve performance and recovery, and provided an evidence-based basis for the application of this method in exercise practice.

## Methods

### Protocol Registration

This systematic review and meta-analysis was performed according to the guidelines of the Preferred Reporting Items for Systematic Reviews and Meta-analysis (PRISMA) (Moher et al., 2015), which included the procedures of review, search strategy, inclusion and exclusion criteria, quality assessment of included studies, and extraction process and analysis. The review protocol was prospectively registered on PROSPERO (CRD42021253386) and was publicly available at https://www.crd.york.ac.uk/prospero/display_record.php?ID=CRD42021253386.

### Literature Search

A comprehensive literature search was performed using the following electronic databases: EMBASE, CENTRAL, PubMed, Web of Science, and SPORTDiscus. All databases were searched for eligible manuscripts from the date of inception to May 22, 2021. A Boolean search mode was applied using the following keywords (MeSh): “Hyperbaric Oxygenation” OR “Oxygen Inhalation Therapy” OR “Hyperbaric oxygen therapy” OR “Hyperbaric oxygen” OR “HBO_2_” OR “HBOT” OR “OHB” AND “sport^∗^” OR “exercise^∗^.” In addition, further searches were performed using the reference lists of relevant reviews on this topic to ensure that the retrieval of studies was as comprehensive as possible. No restrictions were placed on participant sex in the search and the inclusion criteria. Two review authors screened articles using a double-blinded, standardized, independent approach, and differences in their assessments were resolved through discussions or by a third reviewer.

### Inclusion and Exclusion Criteria

The primary focus of this screening was to identify studies that assessed the effect of exercise performance on HBO_2_ therapy in different phases. All duplicate studies were removed primarily by using the reference management software Endnote (X9.2; Thomson Reuters) and then screened using the included and eligible criteria. Studies were included in this review if they met the following criteria: (1) enrolled healthy adults who were considered physically active; (2) performed HBO_2_ therapy; (3) included a control group exposed to normobaric normoxic (NN) conditions; (4) performed physical testing (isokinetic or dynamic strength exercise, maximal incremental treadmill/cycle exercise, etc.); and (5) included at least one exercise performance/recovery index as an outcome measures.

Studies involving objective conditions with no full text available and those with non-English literature and abstracts were excluded. Studies involving outcome measures for the nervous system were also excluded (Jammes et al., 2003). The primary outcome was between-group differences in the HBO_2_ and NN groups or changes from baseline.

### Data Extraction

Two reviewers independently extracted the relevant data for each trial by using a standardized data-extraction form. Discrepancies were resolved through discussion or by a third reviewer. The extracted data included information regarding the source, country, elevation, participant characteristics, exercise protocol, intervention details (phases, atmospheric pressure, concentration, endurance, and oxygen equipment), and outcome measures. The mean and standard deviation (SD) outcome data were extracted. For studies that met the inclusion criteria, if these values were not present, we attempted to contact the corresponding authors in order to obtain these values. In addition, we estimated raw data from graphs by using WebPlotDigitizer software (v4.2, San Francisco, CA, United States),^1^ which were not presented in tables or text.

### Quality and Risk of Bias Appraisal

The quality and risk of bias were assessed according to the criteria proposed by the Cochrane guidelines (Higgins et al., 2019). The Cochrane risk of bias assessment tool evaluates the included studies for several biases, including: (a) random sequence generation (selection bias), (b) allocation concealment (selection bias), (c) blinding of participants and personnel (performance bias), (d) blinding of outcome assessment (detection bias), (e) incomplete outcome data (attrition bias), (f) selective reporting (reporting bias), and (g) other bias. The grades for each of these categories are displayed as either “low risk” (“+”), “high risk” (“–”) or “unclear risk” (“?”). All the operations were independently assessed by two reviewers by using Review Manager 5.4 software (Copenhagen: The Nordic Cochrane Center), and disagreements regarding the quality and risk of bias were resolved through discussion or by a third reviewer.

### Statistical Analysis

Means and standard deviation (SD) values of the outcome measures of pre- and post-exercise HBO_2_ therapy on performance and recovery were independently extracted for analysis. The mean difference (MD) and 95% confidence interval (95% CI) for continuous outcomes reported at the end of the intervention were extracted for analysis. When possible, the between-study variance for the results in the meta-analysis was measured using a random-effects model. Meta-analysis was performed when study interventions were sufficiently similar to be combined, and the heterogeneity of treatment effects was assessed using the I^2^ statistic, chi-square (*p* < 0.1 considered as significant), and Tau^2^, as outlined in a previous study (Higgins et al., 2003). In a test for I^2^ using a scale of low (<25%), moderate (25–75%), and high (≥ 75%), the associated significance level was *p* < 0.05. All analyses were combined with visual inspection of the forest plot, where we used comprehensive meta-analysis by Review Manager 5.3 (Copenhagen: The Nordic Cochrane Center). When intervention data could not be obtained or quantitative synthesis for meta-analysis was not possible, the results were described narratively. Sensitivity analysis (study-by-study deletion) was performed for quantitative synthesis with moderate or high heterogeneity (≥25%) (Higgins et al., 2019). The funnel plots or Egger’s publication bias plots were not generated for outcomes because fewer than 10 studies were included for all meta-analyses (Higgins et al., 2019).

## Results

**FIGURE 1:**
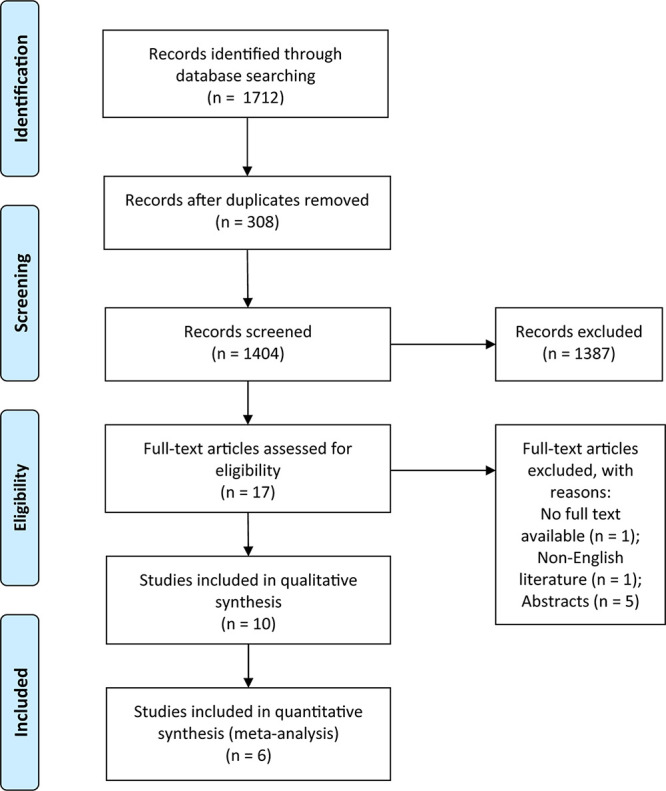
Flow diagram of studies in the systematic review.

A total of 1,712 identified records were screened within the five databases, of which 1,387 duplicates were removed, and 17 articles with full text available were potentially assessed for eligibility. We reviewed the full text of the manuscripts according to the predetermined inclusion and exclusion criteria and included 10 studies enrolling 166 participants in the qualitative analysis and six studies with 69 participants in the quantitative synthesis (meta-analysis). The low number of studies in the quantitative synthesis was primarily due to variations in therapy phases (e.g., pre-exercise, post-exercise, intra-exercise) and outcomes (e.g., blood lactate concentration, creatine kinase, lactate dehydrogenase, maximum oxygen uptake, peak power output, etc.), which prevented meta-analysis. The PRISMA diagram in Figure 1 summarizes the results of the screening and selection process of the study.

### Study Characteristics

The comprehensive characteristics of the studies included in the systematic review are shown in Table 1. All experiments were conducted at plain elevation in eight different countries. A total of 166 participants (149 males, 17 females) were evaluated in 10 studies, and most studies included small samples (≤20 participants), with the exception of the study by Stewart et al. (2011) which included 55 participants. Four studies evaluated the effects of HBO_2_ therapy intra-exercise on exercise performance (Stewart et al., 2011; Shimoda et al., 2015; Zinner et al., 2015; Burgos et al., 2016). The remaining studies assessed the effects of pre- and post-exercise HBO_2_ therapy on performance and recovery, which included Hodges et al. (2003), experiments 1 and 2 in Rozenek et al. (2007); Kawada et al. (2008), experiment 1 in Branco et al. (2016) and Park et al. (2018), experiment 2 in Park et al. (2018), and Woo et al. (2020), and the meta-analysis included data from these six studies enrolling 69 participants. During the therapy, oxygen pressure ranged from 1.0 to 2.5 ATA in all the trials, and the majority of trials involved a 100% increase in oxygen concentration, except for Hodges et al. (2003) (90%). Most trials included participants exposed to NN conditions as the control group, except three trials from two studies, which implemented participants receiving 1.2 ATA and 20.9% higher oxygen concentration as the control groups (Rozenek et al., 2007; Shimoda et al., 2015). The effect of HBO_2_ in improving exercise performance was assessed using the exercise protocol of the maximal incremental test in five studies (Hodges et al., 2003; Burgos et al., 2016; Park et al., 2018; Woo et al., 2020) and experiment 1 of Rozenek et al. (2007), dynamic strength exercises in four studies (Kawada et al., 2008; Stewart et al., 2011; Shimoda et al., 2015) and experiment 2 of Rozenek et al. (2007), and simulated specific training in two studies (Zinner et al., 2015; Branco et al., 2016).

**TABLE 1:** Characteristics of the included studies.

**Study**	**Country**	**Exp**	**Participants**	**Elevation**	**Exercise Protocol**	**HBO_2_ set-up**				**Outcome measure**
						**Phases**	**Experimental**	**Control**	**Equipment**	
Branco et al. (2016)	Brazil	1	11 experienced male adult jiu-jitsu athletes; Mean ± SD age, 29.7 ± 6.6 years	Plain	Training sessions, lasted for 1 h and 30 min	Post-exercise	2.39 ATA, 100% oxygen concentration, 89 min	NN, 90 min	Multiplace Fogliene^®^ (FH 220-5, Brazil)	Bla, RPE, CK, LDH, RPR, Cortisol, Testosterone, AST, ALT
Burgos et al. (2016)	Chile	1	12 young male soccer players; Mean ± SD age, 18.6 ± 1.6 years	Plain	Maximal incremental cycling test, started 75W, the workload increased 25 W⋅min^–1^	Intra-exercise (3 weeks endurance exercise on a cycle ergometer)	2.0 ATA, 100% oxygen concentration,	NN	Hyperbaric chamber (C.H.10 N°4, Osorio Hnos. y Cia. Ltda., Chile)	Bla, VO_2m__ax_, PPO, PO_2_
Hodges et al. (2003)	Canada	1	10 trained male volunteers; Mean ± SD age, 25.7 ± 5.5 years	Plain	Maximal incremental treadmill test, started 5 mph (134 m/min) and 5% grade, speed increased by 0.5 mph (13.4 m/min) ⋅min^–1^	Pre-exercise	2.5 ATA, 90% oxygen concentration, 90 min	NN, 90 min	Sigma Plus monoplace hyperbaric chamber (Perry Baromedical Corporation, Riviera, Florida, United States)	Bla, HR_max_, RPE, TTF, VO_2m__ax_, Venous PO_2_, tcPO_2_
Kawada et al. (2008)	Japan	1	6 men who had been undergoing HIRT for 1 year or more; Mean ± SD age, 26.0 ± 3.9 years	Plain	MVC of knee extensors, 30 repetitions 3 × 2 sets	Pre-exercise	1.3 ATA, 100% oxygen concentration, 50 min	NN, 50 min	Hyperbaric chamber (Shenpix Hyperbaric Medical Trainer, SHENPIX Co., Ltd., Maebashi, Japan)	Bla, isometric knee extensor torque, iEMG, Fatigue index
Park et al. (2018)	Korea	1	10 healthy male amateur soccer players; Mean ± SD age, 21.0 ± 1.25 years	Plain	Maximal incremental treadmill test, Bruce protocol	Pre-exercise	1.3 ATA, 100% oxygen concentration, 30 min	NN, 30 min	Saebo Energy (SB-153 ultimate, Seoul, South Korea)	Bla, HR_max_
		2	10 healthy male amateur soccer players; Mean ± SD age, 21.0 ± 1.25 years	Plain	Maximal incremental treadmill test, Bruce protocol	Post-exercise	1.3 ATA, 100% oxygen concentration, 30 min	NN, 30 min	Saebo Energy (SB-153 ultimate, Seoul, South Korea)	BAP
Rozenek et al. (2007)	America	1	10 healthy male subjects who were considered physically active and exercised at least 3 times a week; Mean ± SD age, 28.0 ± 2.8 years	Plain	Maximal incremental treadmill test, speed was held constant at 268 m⋅min^–1^	Pre-exercise	2.0 ATA, 100% oxygen concentration, 60 min	1.2 ATA, 20.9% oxygen concentration, 60 min	Monoplace hyperbaric chamber (2500 B, Sechrist Industries, Santa Ana, CA)	Bla, HR_max_, RPE, TTF
		2	10 healthy male subjects who were considered physically active and exercised at least 3 times a week (different from exp 1); Mean ± SD age, 23.2 ± 2.4 years	Plain	TTF at 30% of MVC of bench press	Pre-exercise	2.0 ATA, 100% oxygen concentration, 60 min	1.2 ATA, 20.9% oxygen concentration, 60 min	Monoplace hyperbaric chamber (2500 B, Sechrist Industries, Santa Ana, CA)	Bla, HR_max_, RPE, TTF
Shimoda et al. (2015)	Japan	1	20 healthy males; Mean ± SD age, 22.0 ± 1.1 years	Plain	FT, Maximal voluntary unilateral isometric plantar flexions, 50 times × 2 sets	Intra-exercise (between two FT)	2.5 ATA, 100% oxygen concentration, 60 min	1.2 ATA, 20.9% oxygen concentration, 70 min	Multiperson hyperbaric chamber (NHC-412-A; Nakamura Tekkosyo, Ibaraki, Japan)	MVC torque, Electromyography indexes
Stewart et al. (2011)	Netherland	1	55 healthy volunteers; mean age = 26 years, range 23–30 years	Plain	Maximal grip contraction for 1 min × 2 sets, 30 s intervals of recovery	Intra-exercise (performed whole protocol)	2.5 ATA, 100% oxygen concentration, 5 min	NN, 30 min	Four-person multiplace 5500-square-foot Class I hyperbaric chamber (Clucas Diving, Ltd.)	Maximal initial (recovery) grip, time to 50% of max, total effort
Woo et al. (2020)	Korea	1	12 healthy males; Mean ± SD age, 21.67 ± 2.34 years (experimental, *n* = 6), 23.67 ± 3.44 years (control, *n* = 6)	Plain	Maximal incremental treadmill test, Bruce protocol	Post-exercise	2.5 ATA, 100% oxygen concentration, 20 min × 3 sets, a 5 min break between every set	NN, 60 min	Multi-pressure chamber (Interocean I.O Medical, Busan, South Korea)	BAP, CK, LDH, d-ROMs
Zinner et al. (2015)	Germany	1	10 male cross-country skiers and triathletes; Mean ± SD age, 25.3 ± 4.1 years	Plain	Three 3-min simulated double-poling sprints on a cross-country ski ergometer, 3-min intervals of recovery	Intra-exercise (each recovery period)	1.0 ATA, 100% oxygen concentration, 3 min	NN, 3 min	A 170 L Douglas Bag (Hans Rudolph Inc., Shawnee, KS, United States)	Bla, RPE, PPO, PO_2_, SaO_2_, TSI

*Exp: experiment; HBO_2_, hyperbaric oxygen; ATA, absolute atmosphere; NN, normobaric normoxic; Bla, blood lactate concentration; RPE, rating of perceived exertion; CK, creatine kinase; LDH, lactate dehydrogenase; VO_2m__*ax*_, maximum oxygen uptake; PPO, peak power output; PO_2_, partial pressure of oxygen; HR_*max*_, peak heart rate; TTF, time to task failure; HIRT, high-intensity resistance training; MVC, maximal voluntary contraction; BAP, biological antioxidant potential; FT, fatigue test; SaO_2_, arterial oxygen saturation; iEMG, integral electromyogram; RPR, rating of perceived recovery; ALT, alanine aminotransferase; AST, aspartate aminotransferase; d-ROMs, derivatives of reactive oxygen metabolites; tcPO_2_, transcutaneous oxygen tension; TSI, tissue saturation index.*

### Main Findings

#### The Effects of Pre-exercise HBO_2_ Therapy on Exercise Performance

**FIGURE 2:**
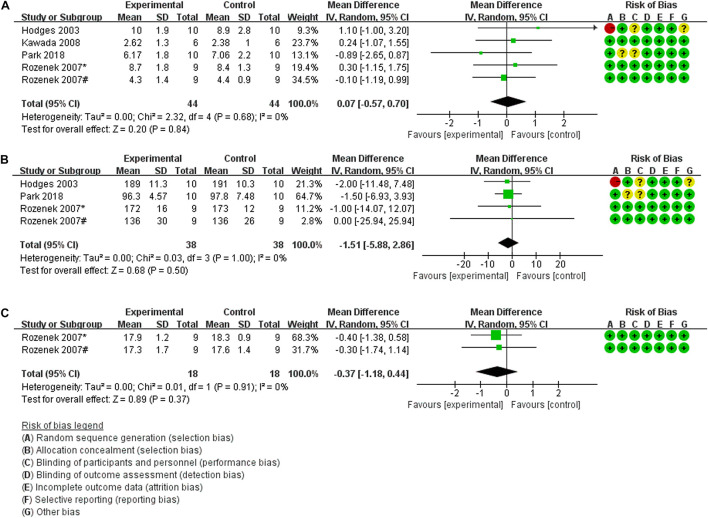
Forest Plot of the Comparison of the effects of pre-exercise HBO_2_ on exercise performance. **(A)** Blood lactate concentration index, **(B)** peak heart rate index, **(C)** rating of perceived exertion index; *: experiment 1; #: experiment 2.

Five trials (46 participants) evaluated blood lactate concentration (Bla) as an outcome measure of the effects of pre-exercise HBO_2_ therapy on exercise performance. The findings of the meta-analyses demonstrated that although the heterogeneity was low (Chi^2^ = 2.32, *P* = 0.68, *I*^2^ = 0%), there was no significant difference (MD: 0.07, [95% CI: –0.57, 0.70], *Z* = 0.20, *P* = 0.84) between HBO_2_ and NN (Figure 2A). For the outcome measure of peak heart rate (HR_max_), the meta-analyses of four trials (40 participants) are presented in Figure 2B; the findings showed no heterogeneity (Chi^2^ = 0.03, *P* = 1.00, *I*^2^ = 0%) and no statistical significance (MD: –1.51, [95% CI: –5.88, 2.86], *Z* = 0.68, *P* = 0.50). Only two trials (20 participants) by Rozenek et al. (2007) measured the rating of perceived exertion (RPE), and found no significant effect of HBO_2_ on RPE (MD: –0.37, [95% CI: –1.18, 0.44], *Z* = 0.89, *P* = 0.37), although the heterogeneity was low (Chi^2^ = 0.01, *P* = 0.91, *I*^2^ = 0%) (Figure 2C).

Furthermore, three of these four studies used pre-exercise HBO_2_ therapy to evaluate subsequent performance, but their results could not be quantitatively synthesized in part because of different outcome measures. Hodges et al. (2003) showed no significant differences in VO_2m__ax_ between the baseline condition and HBO_2_ therapy (MD: –0.30, 95% CI: –5.56, 4.96). Kawada et al. (2008) collected several indices of muscle strength (isometric knee extensor torque, iEMG, fatigue index) and found that the torque during the first half of the first set was significantly low between NN and HBO_2_, while the rest of the experimental results did not show any significant differences. Second, due to different types of exercise testing (Table 1), the time to task failure (TTF) index, which was measured by Hodges et al. (2003) and Rozenek et al. (2007) could not be quantitatively synthesized, and their respective results were not statistically significant (MD: 0.00, [95% CI: –1.92, 1.92]; (MD: –2.71, [95% CI: –16.04, 10.61], respectively).

#### The Effect of Post-exercise HBO_2_ Therapy on Recovery

**FIGURE 3:**
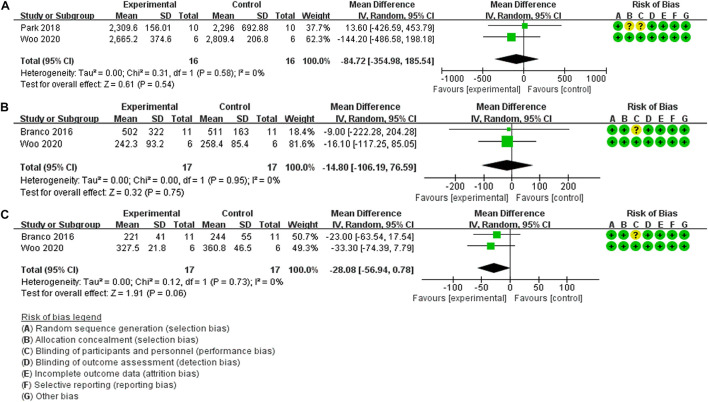
Forest Plot of the Comparison of the effects of post-exercise HBO_2_ on recovery. **(A)** Biological antioxidant potential index, **(B)** creatine kinase index, **(C)** lactate dehydrogenase index.

Evaluation of biological antioxidant potential (BAP) as an outcome measure of functional recovery of exercise performance after HBO_2_ therapy was performed in two trials (22 participants) (Park et al., 2018; Woo et al., 2020). The findings of the meta-analyses demonstrate that although the heterogeneity was low (Chi^2^ = 0.31, *P* = 0.58, *I*^2^ = 0%), there was no significant difference (MD: –84.72, [95% CI: –354.98, 185.54], *Z* = 0.61, *P* = 0.54) between HBO_2_ and NN (Figure 3A). For the outcome measure of creatine kinase (CK) level, the results of meta-analyses of two trials (23 participants) (Branco et al., 2016; Woo et al., 2020) are presented in Figure 3B; the findings indicated no heterogeneity (Chi^2^ = 0.00, *P* = 0.95, *I*^2^ = 0%), and no statistical significance (MD: –14.80, [95% CI: –106.19, 76.59], *Z* = 0.32, *P* = 0.75). Similarly, Branco et al. (2016) and Woo et al. (2020) also investigated the effects of post-exercise HBO_2_ therapy on recovery by using the outcome measure of lactate dehydrogenase (LDH) level, and the results of quantitative synthesis showed no heterogeneity (Chi^2^ = 0.12, *P* = 0.73, *I*^2^ = 0%), with no statistical significance (MD: –28.08, [95% CI: –56.94, 7.79], *Z* = 1.91, *P* = 0.06) (Figure 3C).

Additionally, three studies on the effect of post-exercise HBO_2_ therapy on recovery could not be quantitatively synthesized due to the differences in outcome measures. Branco et al. (2016) measured Bla, RPE, rating of perceived recovery (RPR), hormonal responses, and cellular damage indices. For evaluations using RPR, there was a positive effect between HBO_2_ and NN at 2 h and 24 h post-injection (*Z* = 2.52, *P* = 0.012, *r* = 0.76, and *Z* = 2.37, *P* = 0.018; *r* = 0.71, respectively). For Bla or RPE, there was no difference between the two therapy conditions (MD: –1.70, [95% CI: –13.55, 10.15]; (MD: 0.00, [95% CI: –1.32, 1.32], respectively). The remaining indexes (alanine aminotransferase, aspartate aminotransferase, cortisol, and testosterone) showed a time effect with higher values at all-time points compared to pre-exercise values, but no statistical significance between groups. Woo et al. (2020) investigated the effect of HBO_2_ therapy on the changes in variables related to oxidative/antioxidant balance by evaluating derivatives of reactive oxygen metabolites (d-ROMs), and the results showed no significant difference (*F* = 0.512, *P* = 0.728) between groups, similar to BAP (*F* = 0.657, *P* = 0.626). In this study (Hodges et al., 2003), venous partial pressure of oxygen (PO_2_) and transcutaneous oxygen tension (tcPO_2_) data were compared between the baseline condition and after HBO_2_ therapy. There was a significant change in venous PO_2_ and tcPO_2_ over time after HBO_2_ treatment (*F* = 6.61, df = 8.40, *P* < 0.001 and *F* = 11.93, df = 1.18, *P* = 0.003, respectively), but the difference was not statistically significant between the groups.

#### The Effect of HBO_2_ Therapy During Exercise on Performance

Four studies analyzed the effect of HBO_2_ therapy during exercise on exercise performance. However, they could only be qualitatively synthesized because of the different outcome measures and detailed exercise protocols. Two of these studies evaluated muscle oxygenation capacity using the outcome measures of Bla, peak power output (PPO), and partial pressure of oxygen (PO_2_). All variables showed significant effects of HBO_2_ therapy on the post-exercise values in the experimental and control groups in comparison with the baseline condition, but no significant significance was observed between the groups, except for the PO_2_ values of HBO_2_ therapy in Zinner et al. (2015) (MD: 252.90, [95% CI: 214.07, 291.73]). Moreover, SaO_2_ and the tissue saturation index (TSI) showed a significant effect between HBO_2_ and NN (*P* < 0.05) in the trial by Zinner et al. (2015). Two other studies applied several outcome measures (MVC torque, electromyography indexes, maximal strength, etc.) to evaluate muscle fatigue in exercise performance. In the study by Stewart et al. (2011), the initial/recovery maximal grip and total 1-min effort showed significant effects (*P* < 0.001; MD: 5.87, [95% CI: 2.01, 9.73]; MD: 5.54, [95% CI: 3.01, 8.07], respectively) of HBO_2_ therapy during exercise. For the initial/recovery maximal grip, the time to reduction to 50% of maximum was significantly shorter with HBO_2_ therapy (*P* < 0.01; MD: –5.20, [95% CI: –7.34, –3.06]; MD: –2.27, [95% CI: –5.45, 0.91], respectively), but in comparison with NN, force production remained at a higher level. In the study by Shimoda et al. (2015), MVC torque values were continuously higher in the HBO_2_ group than in the NN group throughout the test, and were significantly higher during repetitions 41–50 (*P* = 0.049, MD: 5.30, [95% CI: 4.33, 6.27]), but did not show significant differences in the other repetitions. For the EMG signals, the root mean square (RMS) of the soleus (Sol) and medial gastrocnemii (MG) in the fatigue test during repetitions 31–40 and repetitions 41–50, respectively, after HBO_2_ therapy were significantly smaller in comparisons between the HBO_2_ and NN groups (MD: –10.70, [95% CI: –13.13, –8.27], *P* = 0.034; MD: –13.40, [95% CI: –16.07, –10.73], *P* = 0.049, respectively), but no differences were observed for the other repetitions. However, the plantar flexion torque, other electromyography indexes, and M wave in response to electrical stimuli during HBO_2_ therapy were not significantly different between the groups.

### Risk of Bias

**FIGURE 4:**
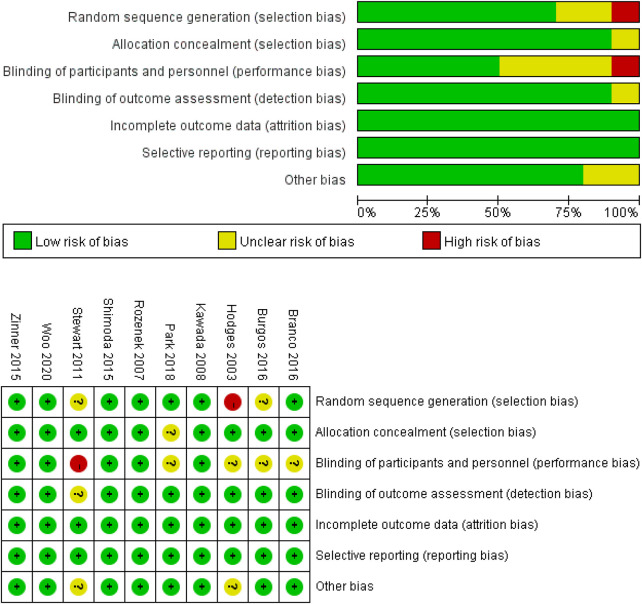
Analysis of the risk of bias in accordance with the Cochrane collaboration guidelines.

We applied Review Manager 5.3 to complete a Cochrane risk of bias assessment. The risk of bias for the effect of HBO_2_ in improving exercise performance was deemed low in most studies. The risk of bias was low for the majority of the studies in evaluations performed by the tool, with five studies showing low risks of biases related to randomization (Stewart et al., 2011; Burgos et al., 2016), allocation concealment (Park et al., 2018), blinding of participants (Hodges et al., 2003; Branco et al., 2016; Burgos et al., 2016; Park et al., 2018), and outcome assessment (Stewart et al., 2011), and an unclear risk for other biases (Hodges et al., 2003; Stewart et al., 2011). Furthermore, the studies judged as having a high risk of bias for “random sequence generation” and “blinding of participants and personnel” were reported by Hodges et al. (2003) and Stewart et al. (2011), respectively. The risk of bias graph and summary are shown in Figure 4.

## Discussion

To our knowledge, this is the first systematic review and meta-analysis to evaluate the effectiveness of HBO_2_ therapy in improving exercise performance and recovery in healthy adults. This systematic review included 10 studies with 166 participants that examined the effects of HBO_2_ therapy on exercise performance, and its purpose was to identify the effects of therapy administered in different phases. The principal findings of the review were as follows: (1) pre-exercise HBO_2_ therapy appears to have no significant effect on subsequent exercise performance in comparison to the control group; (2) post-exercise HBO_2_ therapy during the recovery phase did not affect muscle damage or physiological responses in comparison with an NN environment; (3) HBO_2_ therapy during exercise appears to induce an effective improvement in muscle oxygenation and muscle fatigue.

The beneficial effects of hyperoxia supplementation on oxygen transport system capacity, lactic acid metabolism capacity, power output performance, and endurance exercise tolerance have been described in multiple previous studies (Sperlich et al., 2017; Cardinale and Ekblom, 2018). With regard to exercise performance, Knight et al. (1993) used a combination of femoral arterial and venous measurements of blood flow to show that HBO_2_ treatment could increase the VO_2m__ax_ of the exercising leg, and three other studies showed that hyperoxic conditions can enhance performance (Cunningham, 1966; Ekblom et al., 1975; Wilson and Welch, 1975). Likewise, BLA, HR_max_, and RPE are commonly used monitoring indices in training practice and an effective basis for evaluating training intensity, motor ability, and metabolic function (Glass et al., 1992; Weinstein et al., 1998; Eston, 2012). The absence of a significant effect in our quantitative synthesis thus seems to be at odds with some of the previous research. However, none of the previous studies were RCTs, nor were they design-blind, and all were conducted before the 1980s, necessitating a rational approach to the evaluation of their experimental rigor and conclusions. Horrigan et al. (1979) showed that muscle PO_2_ decreased exponentially after HBO_2_ exposure; therefore, the time interval between the end of HBO_2_ exposure and the start of exercise was relevant in these analyses. These four studies in the quantitative analysis shortened the time between the two events as much as possible for experimental design considerations; however, these investigators did not measure the tissue and blood PO_2_. Furthermore, two studies indicated that pre-exercise HBO_2_ did not enhance aerobic performance (Webster et al., 1998; McGavock et al., 1999). The ergogenic effect of HBO_2_ was not obvious, because under HBO_2_ conditions, the increased dissolved oxygen content was released immediately instead of being released in a timely manner to enhance aerobic exercises. For the slight improvement in HBO_2_ therapy with isometric knee extensor torque pre-exercise (Kawada et al., 2008), the possible mechanism is that hyperoxic gas supplementation can increase the activity of neurons and keep motor units in a stable state of activation during intense exercise, thus reducing peripheral fatigue and delaying muscle contraction fatigue (Cardinale and Ekblom, 2018). The differences in outcome indicators among the included studies may be related to the individual status of participants in the study, exercise intensity, exercise duration, and other factors. Accordingly, future studies should adopt sports performance indicators close to the specific technical movements of different sports, and on this basis, conduct a rigorous RCT.

In the section titled *The effect of post-exercise HBO_2_ therapy on recovery* (Figure 3), there was no statistical difference in BAP, CK, and LDH levels between HBO_2_ and NN in the quantitative synthesis. Reactive oxygen species (ROS) are produced due to incomplete reduction of oxygen during intensive exercise, which leads to a continuous increase in oxidative stress (OS) levels (Powers and Jackson, 2008), necessitating a greater supply of oxygen. Additionally, the levels of serum BAP serve as an important indicator of exercise-induced OS and can directly reflect the OS level and antioxidant capacity. Specifically, existing research has reported that BAP levels increase significantly after the maximal incremental treadmill test (Sugita et al., 2016), mountain bike exercise (Martarelli and Pompei, 2009), and cycling at 75% VO_2m__ax_ intensity (Aoki et al., 2012). Thus, the BAP indexes of the two studies (Park et al., 2018; Woo et al., 2020) were quantitatively synthesized to reveal the effect of HBO_2_ therapy on recovery. The negative conclusion of the meta-analysis could be due to the high antioxidant activity of participants who were trained for exercise over time (Park et al., 2018) and the higher VO_2m__ax_ levels of the participants (48.20 ± 2.19 mL/kg/min) (Woo et al., 2020) in comparison with their peers (Thompson et al., 2013), which led to higher levels of serum BAP at rest (2348.95 ± 266.02 μmol/L, 2647.61 ± 245.45 μmol/L, respectively) in comparison with those reported in previous studies (from 1938.5 to 2347.3 μmol/L) (Martarelli and Pompei, 2009; Aoki et al., 2012; Sugita et al., 2016). However, high-intensity exercise may be one of the main causes of increased OS, and future studies are needed to more fully demonstrate the recovery effect of post-exercise HBO_2_ therapy by analyzing serum ROS levels as well as superoxide dismutase (SOD) and catalase levels. Among the markers of cell damage, CK and LDH are important blood indicators for assessing muscle injury after intensive exercise, and can also be used for fatigue monitoring during the recovery period after exercise (Zajac et al., 2014; Nowakowska et al., 2019). HBO_2_ therapy during the post-exercise recovery phase can effectively alleviate exercise-induced muscle injury (Branco et al., 2016; Woo et al., 2020). The negative conclusion in this meta-analysis probably occurred because the intensity of the exercise did not adequately break down the muscle tissue, limiting the expression of serum CK and LDH levels and the subsequent recovery effect. Therefore, in the recovery stage after exercise, no significant difference was observed between the HBO_2_ and NN groups. Meanwhile, serum CK and LDH levels tended to increase within 72 h after exercise. Thus, considering the half-life of CK and LDH, additional studies are needed to verify the effect of HBO_2_ on fatigue recovery.

RPR is an important indicator of human sensory recovery and is of particular significance for evaluating the effect of fatigue recovery (Laurent et al., 2011). Branco et al. (2016) showed that HBO_2_ therapy improved RPR, which is consistent with the findings reported in another study (Kim et al., 2011). However, with varying RPR being the only significant difference in this part of the systematic review, attention should be paid to the potential influence of the placebo effect, as demonstrated by previous research (Broatch et al., 2014; Pinho Júnior et al., 2014). PO_2_ and/or tcPO_2_ are reliable assessments of tissue oxygen availability (Sheffield, 1998). HBO_2_ therapy improves the respiration efficiency of mitochondria, increases the partial pressure of oxygen in blood vessels, improves the dispersion of oxygen in capillaries, and improves the oxygen transport capacity of the body (Sperlich et al., 2017; Cardinale and Ekblom, 2018; Shankar et al., 2018). However, data obtained by Hodges et al. (2003) showed that plasma and tissue oxygen levels did not increase after HBO_2_ therapy. This was probably because the oxygen in the blood was mainly transported by binding to hemoglobin, only a small part of which was directly dissolved, and the hemoglobin levels in the body were stable (Wajcman and Kiger, 2002; Klotz, 2003). The data also support the rationale that HBO_2_ therapy does not improve exercise performance (Hodges et al., 2003).

Burgos et al. (2016) suggested that antioxidant capacity did not improve during 3 weeks of HBO_2_ training, training in an HBO_2_ environment did not change oxidative stress in volunteers, and that changes in cellular antioxidant defenses may mediate these results (Radak et al., 2008). Thus, future studies should aim to determine the mechanism of this response to HBO_2_. The balance between oxygen supply and consumption was tested using the indicators PO_2_, S_a_O_2_, and TSI (Zinner et al., 2015), which indicates that intermittent oxygen supplementation can improve muscle oxygenation level during high-intensity interval training. HBO_2_ treatment during exercise conditions may have increased alveolar-capillary oxygen exchange (Weaver et al., 2009; Casey et al., 2011); therefore, further research is required to clarify this aspect. Stewart et al. (2011) confirmed that short, sustained exposure to HBO could enhance the ability of forearm muscles to generate force during maximum sustained contractions. One possible mechanism for fatigue during reduced muscle oxygenation may be an increase in intracellular ADP, which may be associated with the inhibition of cross-bridge dissociation and, therefore, actin movement during muscle contraction (Debold et al., 2008). Studies have shown that an increase in intracellular inorganic phosphate (Pi) during muscle contraction may lead to muscle fatigue by limiting the release of Ca^++^ from the sarcoplasmic reticulum (Westerblad et al., 2002), and increased tissue oxygen tension prior to muscle contraction may inhibit intracellular ADP and/or Pi concentrations and promote PCr synthesis of ATP, resulting in a cross-bridge closed loop (Stewart et al., 2011). This could explain why sustained HBO_2_ therapy maintains power output at a higher level throughout muscle contraction. According to Shimoda et al. (2015), MVC torque values, RMS, and MG were continuously higher in the HBO_2_ group than in the NN group throughout the repetitive movement (50 repetitions) (during repetitions 41–50; during repetitions 31–40; during repetitions 41–50, respectively). These results indicate that during exercise, HBO_2_ therapy could inhibit the progression of muscle fatigue and bring about sustained output of muscle strength, but the effect on short-term maximum strength generation was not obvious (Shimoda et al., 2015). Reductions in the motor unit complement, synchronicity, and firing rates were considered to be the main reasons for the decrease in the RMS of EMG signals in active muscle groups during maximum contraction exercise induced by muscle fatigue (Kaufman et al., 1984; Crenshaw et al., 2000). During exercise, HBO_2_ therapy could increase the synchronization and firing rates of motor units (Gosselin et al., 2004), and then improve the activity ability of neurons, so that motor units can maintain a stable activation state during high-intensity exercise, and then reduce the degree of peripheral fatigue degree (Cardinale and Ekblom, 2018). Because the mechanism by which HBO_2_ affects muscle recovery from fatigue is unclear, it is necessary to investigate the benefits of HBO_2_ therapy for athletes in greater depth.

In this systematic review and meta-analysis, the PRISMA declaration list was strictly followed (Moher et al., 2015), and the included studies were generally high-quality studies, but the current study had some limitations: (a) the inclusion and exclusion criteria were designed to allow enrollment of healthy adults who were considered physically active; as a result, the number of studies included in the systematic review was insufficient; (b) the exercise protocols differed among studies (e.g., maximal incremental cycling test, TTF at 30% of MVC of bench press; MVC of knee extensors, etc.); (c) the studies used a variety of oxygen equipment and oxygen dosages (Table 1); (d) most of the studies were small and only investigated male participants, except Stewart et al. (2011); (e) and the conclusions of qualitative analysis needed to be confirmed by a large number of further empirical studies.

## Conclusion

This systematic review and meta-analysis clearly indicated that pre-exercise HBO_2_ therapy had no significant effect on subsequent exercise performance, and the effect of post-exercise HBO_2_ therapy on recovery was not obvious. However, HBO_2_ therapy administered during exercise can improve muscle endurance performance. Despite the limitations discussed above, this systematic review included studies describing exercise protocols of various types and durations as well as HBO_2_ equipment with various set-ups and oxygen dosages; therefore, our conclusions, including the dose-effect relationship between exercise design and oxygen supplementation, need to be confirmed by a large number of follow-up studies and more abundant evidence-based evidence should be obtained.

## Data Availability Statement

The original contributions presented in the study are included in the article/supplementary material, further inquiries can be directed to the corresponding author/s.

## Author Contributions

XH and RW participated in the study design and drafted the manuscript. XH and ZZ were responsible for writing the manuscript. BG, GW, and RW participated in the overall editing and approval of the manuscript. BG was in charge of financial support. All authors contributed to the article and approved the submitted version.

## Conflict of Interest

The authors declare that the research was conducted in the absence of any commercial or financial relationships that could be construed as a potential conflict of interest.

## Publisher’s Note

All claims expressed in this article are solely those of the authors and do not necessarily represent those of their affiliated organizations, or those of the publisher, the editors and the reviewers. Any product that may be evaluated in this article, or claim that may be made by its manufacturer, is not guaranteed or endorsed by the publisher.

## Footnotes

1
https://apps.automeris.io/wpd/
